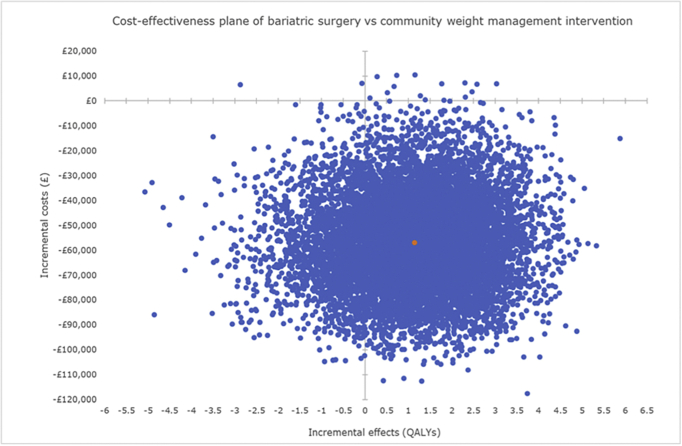# Cost-effectiveness of bariatric surgery versus community weight management to treat obesity-related idiopathic intracranial hypertension: evidence from a single-payer healthcare system

**DOI:** 10.1016/j.soard.2021.03.020

**Published:** 2021-07

**Authors:** Laura Elliot, Emma Frew, Susan P. Mollan, James L. Mitchell, Andreas Yiangou, Zerin Alimajstorovic, Ryan S. Ottridge, Ben R. Wakerley, Mark Thaller, Olivia Grech, Rishi Singhal, Abd A. Tahrani, Mark Harrison, Alexandra J. Sinclair, Magda Aguiar

**Affiliations:** a Department of Economics and Related Studies, University of York, York, United Kingdom; b Institute of Applied Health Research, University of Birmingham, Birmingham, United Kingdom; c Birmingham Neuro-Ophthalmology, University Hospitals Birmingham NHS Foundation Trust, Queen Elizabeth Hospital, Birmingham, United Kingdom; d Department of Neurology, University Hospitals Birmingham NHS Foundation Trust, Queen Elizabeth Hospital, Birmingham, United Kingdom; e Metabolic Neurology, Institute of Metabolism and Systems Research, College of Medical and Dental Sciences, University of Birmingham, Birmingham, United Kingdom; f Centre for Endocrinology, Diabetes and Metabolism, Birmingham Health Partners, Birmingham, United Kingdom; g Institute of Metabolism and Systems Research, College of Medical and Dental Sciences, University of Birmingham, Birmingham, United Kingdom; h Birmingham Clinical Trials Unit, College of Medical and Dental Sciences, University of Birmingham, Birmingham, United Kingdom; i Department of Neurology, Gloucestershire Hospitals NHS Foundation Trust, Gloucester, United Kingdom; j Upper GI Unit and Minimally Invasive Unit, Birmingham Heartlands Hospital, University Hospitals Birmingham NHS Foundation Trust, Birmingham, United Kingdom; k Institute of Cancer and Genomic Sciences, University of Birmingham, Birmingham, United Kingdom; l Department of Endocrinology and Diabetes, University Hospitals Birmingham NHS Foundation Trust, Birmingham, United Kingdom; m Collaboration for Outcomes Research and Evaluation, Faculty of Pharmaceutical Sciences, University of British Columbia, Vancouver, Canada; n Centre for Health Evaluation and Outcome Sciences, St. Paul’s Hospital, Vancouver, Canada

**Keywords:** Cost-effectiveness, Bariatric surgery, Idiopathic intracranial hypertension, Weight loss

## Abstract

**Background:**

Idiopathic intracranial hypertension (IIH) is associated with significant morbidity, predominantly affecting women of childbearing age living with obesity. Weight loss has demonstrated successful disease-modifying effects; however, the long-term cost-effectiveness of weight loss interventions for the treatment of IIH has not yet been established.

**Objectives:**

To estimate the cost-effectiveness of weight-loss treatments for IIH.

**Setting:**

Single-payer healthcare system (National Health Service, England).

**Methods:**

A Markov model was developed comparing bariatric surgery with a community weight management intervention over 5-, 10-, and 20-year time horizons. Transition probabilities, utilities, and resource use were informed by the IIH Weight Trial (IIH:WT), alongside the published literature. A probabilistic sensitivity analysis was conducted to characterize uncertainty within the model.

**Results:**

In the base case analysis, over a 20-year time horizon, bariatric surgery was “dominant,” led to cost savings of £49,500, and generated an additional 1.16 quality-adjusted life years in comparison to the community weight management intervention. The probabilistic sensitivity analysis indicated a probability of 98% that bariatric surgery is the dominant option in terms of cost-effectiveness.

**Conclusion:**

This economic modeling study has shown that when compared to community weight management, bariatric surgery is a highly cost-effective treatment option for IIH in women living with obesity. The model shows that surgery leads to long-term cost savings and health benefits, but that these do not occur until after 5 years post surgery, and then gradually increase over time.

## Highlights

1. Weight loss is a disease modifying therapy for IIH.
2. This is the first paper to report the cost-effectiveness of IIH weight loss therapies
3. Surgery ‘dominates’ (less costs, more QALYs) community weight management
4. The cost-effectiveness of surgery increases with time.

Idiopathic intracranial hypertension (IIH) is a highly incapacitating condition, characterized by raised intracranial pressure (ICP) [1,2], that mainly affects women living with obesity [3]. Elevated ICP can lead to swelling of the optic nerve head, referred to as papilledema, which can potentially result in blindness [4]. IIH is typically associated with significant morbidity due to chronic, disabling headaches [5] and varying degrees of visual disturbances that cause a reduction in health-related quality of life (HRQoL) and productivity loss in the workplace [6].

The pathogenesis of IIH remains unclear [7]; however, it predominately affects females aged between 25 and 36 years, and obesity is a major risk factor [8]. The annual incidence of IIH in female patients increased from 2.5 per 100,000 person-years in 2005 to 9.3 per 100,000 person-years in 2017 [9]. Once considered a rare condition, the burden of IIH is rapidly increasing [3]. Hospital IIH admissions in England have risen by 442% between 2002 and 2014, and associated costs over the same period increased from £9.2 million to £50 million per annum [3]. This rising incidence of IIH is significantly correlated with rising obesity levels [3]. If the IIH incidence continues to increase at the same rate, these annual costs are projected to increase to £462 million by 2030 [3]. These increasing excessive costs have also been shown in the United States, where the total hospital costs per IIH admission in 2007 were 4 times greater than for a population-based per-person admission, with the total economic costs of IIH patients exceeding $444 million [10].

Weight loss is currently the only established disease-modifying therapy for IIH [8,11]. Lifestyle behavioral weight-loss interventions [12] resulting in 15% weight loss are effective in lowering ICP, improving papilledema and visual functions, and decreasing headache frequency and severity, with concomitant reduction in analgesia usage [11], but long-term weight maintenance is challenging, leading to weight regain in the majority of patients [6]. A recent meta-analysis found reports and series that have shown the beneficial clinical effects of bariatric surgery in resolving IIH [13]. Bariatric surgery is safe and is the most effective method of achieving weight loss (25%–30%) that is sustainable over the long term [14], and consequently reducing IIH, but with high upfront costs. Hence, more research is needed to understand the long-term cost-effectiveness of surgery when compared to lifestyle weight loss interventions.

In England, the National Institute for Health and Care Excellence (NICE) currently recommends bariatric surgery for people with a body mass index (BMI) over 40 kg/m^2^ and for people with a BMI over 35 kg/m^2^ who fulfill certain criteria. IIH is not currently considered as a significant co-morbidity associated with obesity that would qualify for bariatric surgery in patients below a BMI of 40 kg/m^2^.

With the goal of informing new treatment recommendations for IIH and guidelines for bariatric surgery, the IIH weight trial (IIH:WT) was designed to compare the efficacy and cost-effectiveness of bariatric surgery and community weight management interventions [15]. The IIH:WT was conducted within England, and therefore within the context of a single-payer healthcare system (National Health Service [NHS]). This paper reports on the economic evaluation by extrapolating the trial data and modeling the long-term cost-effectiveness from the perspective of the healthcare service in England. The gains are captured using quality-adjusted life years (QALYs), which are commonly used to enable judgements about cost-effectiveness. The IIH:WT findings are modeled over 5-, 10-, and 20-year time horizons to assess the long-term cost-effectiveness of bariatric surgery as a treatment for IIH, compared to a community weight management program.

## Methods

### Study design

IIH:WT was a randomized, controlled, parallel-group, multi-center trial [16]. Inclusion criteria for the trial were female patients with active IIH and no other significant co-morbidity, aged between 18–55 years, with a BMI ≥35 kg/m^2^, consistent with the NICE obesity guideline [17]. The National Research Ethics Committee West Midlands approved the trial (14/WM/0011). The economic evaluation was conducted using a decision-analytic model to facilitate extrapolation of trial findings over an extended time horizon.

### Model structure

**Fig. 1:**
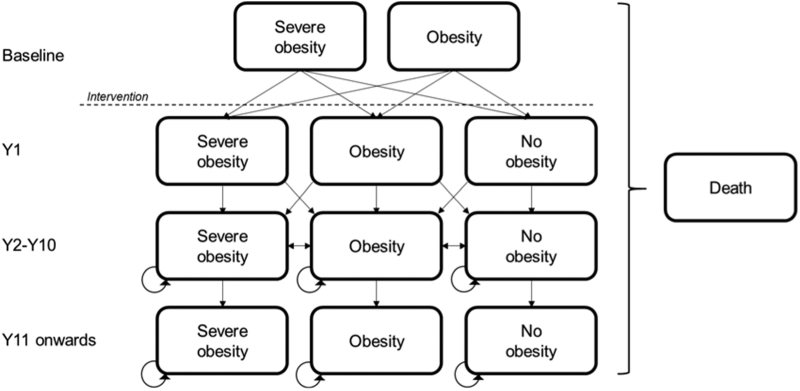
Markov model structure. The structure is idential for both the bariatric surgery arm and the community weight management intervention arm. Arrows indicate possible transitions between states. Patients can transition to “Dead” from any of the states. The dashed line indicates the interventions taking place following baseline measurements.

A Markov model was applied to reflect cyclical fluctuations in weight over time and to allow for the consideration of weight recidivism following bariatric surgery. The model structure is shown in Fig. 1. The model compares bariatric surgery with community weight management, and therefore the surgical arm comprises the suite of surgical procedures that were performed within the IIH:WT. A cycle length of 1 year was used, as this was considered to be the shortest sufficient time period for patients to change between BMI categories. The time horizons for the model were 5, 10, and 20 years.

The model started with a hypothetical cohort of 1000 patients who were considered to have a health state of either obesity or severe obesity, as determined by their BMI and in line with NICE guidance for eligibility for bariatric surgery [17]. Classifying the states according to weight (BMI) category enabled the effects of weight recidivism on IIH status to be captured, as well as allowed for the wider consideration of the health impacts of obesity, such as the incidence of co-morbidities other than IIH.

Patients were distributed between the baseline health states in line with the distribution of BMI from the IIH:WT. Following intervention, the patients then progressed to 1 of 3 states: severe obesity (BMI ≥40 kg/m^2^), obesity (BMI 30–39.9 kg/m^2^), or no obesity (BMI <30 kg/m^2^), or into an absorbing state, death. Maximum weight loss generally occurs between 12–24 months following surgery [18], and from that point onwards, patients typically experience some degree of weight regain. Weight regain continues gradually until approximately 10 years postsurgery, at which point it plateaus [19]. To reflect this process, the model required distinct transition probabilities between health states over 4 time periods: cycle 1, cycle 2, cycles 3–10, and cycle 11 onwards.

### Sources of data

Both primary and secondary data were used to inform the model parameters (Table S1). Data on resource use, costs, and effectiveness were derived from the IIH:WT and supplemented with data from targeted literature review where necessary.

### Transition probabilities

The IIH:WT followed patients for 24 months, and these data were used to estimate the transition probabilities for the first 2 model cycles (Table S2). From year 2 onwards, transition probabilities were calculated using weight regain data from the Swedish Obese Subjects (SOS) study [19], in conjunction with data from the IIH:WT. Once the patients reached cycle 10, their weight was assumed to plateau. Hence, the transition probabilities applied in cycles 11–20 assume only the possibility of transitioning to dead or remaining within the same state. The model included a BMI-specific mortality risk [20] and an additional mortality risk associated with bariatric surgery [21].

### Costs

The costs included in the model were those relevant to the healthcare service, detailed in Table S1. All costs are reported in 2017/2018 GBP prices [22]. For the surgical arm, the design of the IIH:WT did not predetermine the type of surgery to be given, as this was a pragmatic decision to reflect routine clinical practice. The cost of bariatric surgery therefore was determined by the weighted average of the 3 surgery types performed—laparoscopic adjustable gastric band (LAGB), laparoscopic sleeve gastrectomy (LSG), and Roux-en-Y gastric bypass (RYGB)—as the intention of the model was not to recommend a surgery type, but rather to assess the incremental cost-effectiveness of surgery versus community weight management. The total cost of surgery was inclusive of the weighted costs of revisional surgery for gastric banding. Data on resource use derived from the IIH:WT were used in conjunction with the NHS National Tariff [22,23] to calculate the average costs per patient, per cycle for the first 2 cycles. The cost of the community weight management program comprised WeightWatchers vouchers that provided access to online and mobile tools for 12 months.

For each health state, the average cost of IIH management was estimated by multiplying the incidence of IIH by the annual per-patient costs of IIH [3]. Evidence suggests that weight regain results in recurrence of IIH within both individuals living with obesity and those with a healthy weight [6,24,25]. It was therefore assumed that IIH would recur at the same rate regardless of whether the patient had lost weight and then subsequently regained weight or remained in an obesity health state throughout.

### Outcomes

Utility values for each health state were derived from the IIH:WT data (Table S1). HRQoL was assessed using the EuroQoL-5 dimension-5 level (EQ-5D-5L) instrument at baseline and at 12 and 24 months, and it was assumed that utilities remained constant for each health state over time.

All costs and benefits were discounted at 3.5%, consistent with UK NICE guidelines.

### Cost-utility analysis

The cost-utility analysis estimated the cost of the change in QALYs due to surgery when compared with that of the weight management program. This results in an incremental cost-effectiveness ratio, which is the difference in costs divided by the difference in QALYs and gives an estimate of the cost per QALY gained.

### Scenario and sensitivity analysis

To assess the uncertainty around the model parameters, a probabilistic sensitivity analysis was conducted. The probability that surgery is cost-effective when compared to weight management is then estimated for different threshold values of willingness to pay for a QALY, presented as a cost-effectiveness acceptability curve.

To account for the wider cost impact from weight loss and lowering the incidence of these co-morbidities associated with obesity, a scenario analysis was conducted that included costs associated with type 2 diabetes and coronary heart disease (CHD). The annual incidences and associated healthcare costs of CHD and type 2 diabetes were obtained [26] and applied to the health states using the same method described above for IIH. Any disutility associated with diabetes and CHD was not applied, as it was felt that this was already captured within the self-reported HRQoL values from the trial data.

## Results

Across all 3 time horizons of 5, 10, and 20 years, bariatric surgery dominates (less cost and more benefit) the community weight management intervention, generating more QALYs with cost savings (Table 1). The extent of dominance increases over time, with more cost savings accumulating the longer patients are followed-up. At 20 years, surgery led to an incremental cost saving of £49,500 and an additional 1.16 QALYs when compared to community weight management. When the additional costs of type 2 diabetes and CHD were considered, these cost savings increased to £54,300.

**Table 1:** Cost-utility analysis

	Time horizon for model						Additional sensitivity analysis			
	5 yr		10 yr		20 yr		Scenario analysis		Probabilistic sensitivity analysis	
	Surgery	WM	Surgery	WM	Surgery	WM	Surgery	WM	Surgery	WM
Total costs	£15,900	£27,400	£29,900	£58,600	£55,100	£112,400	£81,100	£143,900	£65,500	£118,600
Incremental costs		−£11,500		−£28,700		−£57,300		−£62,900		−£53,000
Total QALYs	3.83	3.53	7.62	7.04	14.28	13.12	14.28	13.12	14.26	13.14
Incremental QALYs		.29		.58		1.16		1.16		1.12
ICER∗		−£39,000		-£49,300		−£49,500		£54,300		−£47,200

WM = weight management; QALY = quality-adjusted life years; ICER = incremental cost-effectiveness ratio.

∗ ICER = difference in total costs between surgery versus WM/Difference in total QALYs between surgery versus WM.

**Fig. 2:**
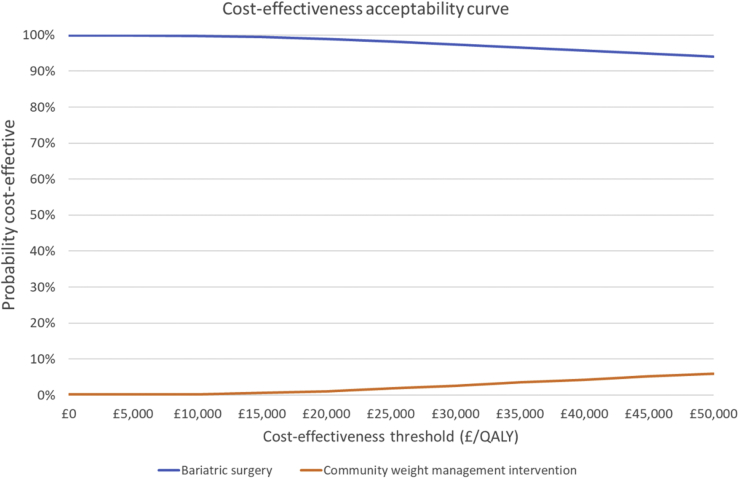
Cost-effectiveness acceptability curve. QALY = quality-adjusted life years.

The probabilistic sensitivity analysis confirmed the base case results, producing a cost saving of £47,200 and an incremental gain of 1.12 QALYs. The distribution of incremental cost-effectiveness ratios is shown in Fig. S1. At a willingness to pay £20,000 per QALY, the lower threshold used by decision-making bodies such as NICE, there is a 98% chance that bariatric surgery is cost-effective when compared to the community weight management intervention (Fig. 2).

## Discussion

This paper is the first to report on the long-term cost-effectiveness of bariatric surgery compared to a community weight management intervention to treat IIH. This was done within the context of a single-payer healthcare system. The economic model combined the costs associated with bariatric surgery to treat IIH with the HRQOL benefits and found it to increase QALYs and decrease costs, when compared to community weight management. This finding was robust to sensitivity analysis. Varying the time horizon showed that both the incremental costs saved and the incremental QALYs gained from surgery increase over time.

Although there is evidence for the cost-effectiveness of bariatric surgery and despite multiple guidelines for treatment, including from NICE [17], access to bariatric surgery within the English healthcare service remains limited, with less than .002% of the potentially eligible adults having surgery annually [27]. This is due to barriers for referral from primary care and funding constraints from the commissioners [27]. This economic model demonstrates that improving access to bariatric surgery is likely to be cost saving, reduce the burden of IIH in women with BMIs ≥35 kg/m^2^, and improve patient HRQoL.

The economic model only included the co-morbidities of type 2 diabetes and CHD as part of a scenario analysis. Expanding the model to account for additional cost savings from reducing the risks of further obesity-related co-morbidities will only make bariatric surgery even more cost saving. Furthermore, the model was conducted from a health service perspective, meaning that any out-of-pocket payments or indirect costs of IIH were not included. If these indirect costs, such as days off work or time to travel to appointments to manage ongoing IIH symptoms, were included, then this would make surgery even more cost saving. The model classified health states according to BMI category with the aim of capturing the wider health impacts of obesity (type 2 diabetes and CHD), as well as effects of weight recidivism on IIH status. A strength of the model is that it used data from the IIH:WT study, including EQ-5D-5L data that were directly collected from patients to construct QALYs. And as the patients moved between the BMI health states over time, the effects of weight loss upon IIH symptoms and wider obesity effects will have been captured within this EQ-5D-5L data and modeled accordingly. Using QALYs as a commensurate outcome allows comparisons of cost-effectiveness to be made between alternative treatments across different disease areas.

There are some limitations to consider. The model uses data from the IIH:WT, and as IIH is a rare condition, the model was unable to differentiate between the different surgery types offered in IIH:WT due to the reduced sample size within each group, so any differential costs and effects were not included. There is evidence that LAGB is associated with lower procedure costs, but has a much higher rate of revisional surgery, as well as a smaller and less well-maintained effect on body weight than RYGB and LSG [28]. However, the range and distribution of bariatric surgeries performed in the IIH:WT broadly reflect current practice in the English healthcare system [15,21], and therefore the results are applicable to assessing surgery versus community weight management for treatment of IIH. At an international level, more research is needed to fully estimate the differential cost-effectiveness between the surgery types. Within the IIH:WT, only half of the community weight management cohort attended 75%–100% of weekly sessions, but evidence suggests this is similar attendance to that seen in other trials [29], and it is unknown how adherence would have varied outside the trial setting. The model used data from the IIH:WT up to the end of cycle 2; beyond that, data from the SOS study were used, as this study contained some of the longest follow-up data available on bariatric surgery. However, this study was conducted 16 years ago, was based exclusively in Sweden, and the surgical techniques differ, as the SOS study also used vertical banded gastroplasty, which is no longer performed; therefore, surgical outcomes may have since changed. Alternative sources of long=term data were available, including a meta-analysis [30]; however, the data were reported as percentages of expected weight loss and the economic model tracked weight trajectories using BMI categories, making it challenging to use these data for the model structure. Within the SOS study, vertical banded gastroplasty weight loss at 10 years was similar to LABG weight loss, and we know that LSG is more effective than LAGB at achieving long-term weight loss; therefore, by using the SOS data to populate the model, we believe this to be a conservative estimate for the likely long-term effectiveness for the surgical arm.

## Conclusion

The results suggest that bariatric surgery is a dominant treatment option for IIH patients living with obesity when compared to a community weight management intervention. It provides evidence to inform funding bodies that IIH should qualify as a co-morbidity of obesity that can be improved with weight loss. Hence, IIH patients with a BMI over 35 kg/m^2^ should meet the criteria to be recommended for bariatric surgery under NICE clinical guidance [17].

## Disclosures

*This Trial was funded by grant NIHR-CS-001-028 (Clinician Scientist Fellowship) from the National Institute for Health Research (Dr Sinclair) and grant MR/K015184/1 from the Medical Research Council UK (Dr Sinclair).* *EF was funded by a National Institute for Health Research (NIHR) career development fellowship award (NIHR-CDF-2015-08-13) for the duration of the study. AJS was funded by an NIHR clinician scientist fellowship (NIHR-CS-011-028) for the duration of the study; is funded by a Sir Jules Thorn Award for Biomedical Science; and reports personal fees from Invex therapeutics, during the conduct of the study but outside the submitted work.* *AAT was funded by an NIHR Clinician Scientist Award for part of the duration of the study (CS-2013-13-029) and reports grants from Novo Nordisk, personal fees from Novo Nordisk, nonfinancial support from Novo Nordisk, personal fees from Eli Lilly, nonfinancial support from Eli Lilly, personal fees from Janssen, personal fees from AZ, nonfinancial support from AZ, nonfinancial support from Impeto medical, nonfinancial support from Resmed, nonfinancial support from Aptiva, personal fees from BI, nonfinancial support from BI, personal fees from BMS, nonfinancial support from BMS, personal fees from NAPP, nonfinancial support from NAPP, personal fees from MSD, nonfinancial support from MSD, grants from Sanofi, and personal fees from Sanofi. SPM reports other Invex Therapeutics, other Heidelberg engineering during the conduct of the study; other from Chugai-Roche Ltd, other from Janssen, other from Allergan, other from Santen, other from Roche, and other from Neurodiem, outside the submitted work. OG reports consulting work for Invex Therapeutics during the conduct of the study but outside the submitted work. BRW reports consulting work for Invex Therapeutics during the conduct of the study but outside the submitted work. All other authors declare no competing interests. The views expressed are those of the authors and not necessarily those of the UK National Health Service, the NIHR, or the UK department of Health and Social Care.*

## Figures and Tables

**Figure S1:**