# Supplementary material for: Cost-effectiveness of bariatric surgery versus community weight management to treat obesity-related idiopathic intracranial hypertension: evidence from a single-payer healthcare system
Source: Surg Obes Relat Dis. 2021 Jul;17(7):1310–6. doi: 10.1016/j.soard.2021.03.020 (PMC8241428; doi:10.1016/j.soard.2021.03.020)
Supplement: Supplementary Material [file mmc1.docx]

**Supplementary Materials**

**Technical detail on model parameters**

**Transition probabilities**

From year 2 onwards, transition probabilities were calculated using weight regain data from the Swedish Obese Subjects (SOS) study(24), in conjunction with data from the IIH:WT trial. The SOS study is a prospective study of patients who underwent bariatric surgery matched with conventionally treated controls. The SOS control group underwent an unstandardised assortment of nonsurgical interventions, which included both sophisticated lifestyle interventions (comprising advice on weight loss through diet and exercise, much like a community weight management programme), and no treatment whatsoever(24). Ten-year follow-up data was available for over 1,700 patients from the SOS study, which included percentage of patients who had experienced a change in BMI category.

These data were used to calculate the BMI for the model cohort from year 3 and at each subsequent year up to 10 years. Health states were then determined for each patient based on their projected BMI, from which transition probabilities were calculated for each cycle up to year 10, in the same manner that they were derived from primary data. During cycles 3-10, it was assumed that all patients had entered the weight recidivism stage.

Hence, the transition probabilities applied in cycles 11-20 assume only the possibility of transitioning to ‘dead’ or remaining within the same state.

A key structural assumption was that transitions were not possible from the ‘severe obesity’ state to ‘no obesity’ state within one cycle, and vice versa, from cycle 1 onwards. This replicates the gradual nature of weight change in the absence of surgical intervention. However, patients could transition from ‘severe obesity’ to ‘no obesity’ between cycle 0 and cycle 1, reflecting the substantial weight loss that may be experienced within a year following bariatric surgery, as observed in the IIH:WT trial. Patients could transition between health states up to cycle 10, after which they were assumed to remain in the same health state, other than the possibility of death. This reflects the minimal variation in weight change beyond 10 years post-surgery(24).

**Utility values**

Due to the absence of any patients in the ‘no obesity’ state in the community weight management intervention arm for the duration of the trial, it was not possible to derive a utility value for this health state from the trial data. A reliable estimate for this value could not be obtained from secondary sources as the proportion of patients within this health state suffering with IIH was unknown. As such, the utility for this health state was assumed to be the same as the corresponding health state within the surgical arm of the model.

**Table S1: Model parameters**

| **Parameter** | **Value** | **Distribution** | **Source** |
| --- | --- | --- | --- |
| **Utilities*** | | | |
| *Bariatric surgery arm* | | | |
| A | 0.6694 | BETA | 21 |
| A1 | 0.6243 | BETA | 21 |
| A2 | 0.7333 | BETA | 21 |
| B | 0.7023 | BETA | 21 |
| B1 | 0.8985 | BETA | 21 |
| B2 | 0.8269 | BETA | 21 |
| C1 | 0.7571 | BETA | 21 |
| C2 | 0.7233 | BETA | 21 |
| *Community weight management intervention arm* | | | |
| A | 0.7410 | BETA | 21 |
| A1 | 0.6335 | BETA | 21 |
| A2 | 0.7671 | BETA | 21 |
| B | 0.7474 | BETA | 21 |
| B1 | 0.7505 | BETA | 21 |
| B2 | 0.6363 | BETA | 21 |
| C1 | 0.7571 | BETA | 21 |
| C2 | 0.7233 | BETA | 21 |
| **Costs** | | | |
| **Unit costs** | | | |
| *Bariatric surgery arm* | | | |
| Bariatric surgery | £6 826 | GAMMA | 21,27 |
| Secondary care costs (baseline) | £184 | GAMMA | 21,27 |
| Primary care costs (baseline) | £120 | GAMMA | 21,27 |
| Prescription med costs (baseline) | £33 | GAMMA | 21,27 |
| Secondary care costs (year 1) | £115 | GAMMA | 21,27 |
| Primary care costs (year 1) | £205 | GAMMA | 21,27 |
| Prescription med costs (year 1) | £17 | GAMMA | 21,27 |
| *Community weight management intervention arm* | | | |
| Community weight management intervention vouchers | £194 | GAMMA | 21 |
| Secondary care costs (baseline) | £125 | GAMMA | 21,27 |
| Primary care costs (baseline) | £188 | GAMMA | 21,27 |
| Prescription med costs (baseline) | £116 | GAMMA | 21,27 |
| Secondary care costs (year 1) | £276 | GAMMA | 21,27 |
| Primary care costs (year 1) | £405 | GAMMA | 21,27 |
| Prescription med costs (year 1) | £47 | GAMMA | 21,27 |
| **Annual disease management costs** | | | |
| Annual management cost of IIH | £8 072 | GAMMA | 4 |
| Annual management cost of diabetes | £4 545 | GAMMA | 35 |
| Annual management cost of CHD | £2 324 | GAMMA | 31 |
| **Health state costs*** | | | |
| *Bariatric surgery arm* | | | |
| A | £4 997 | GAMMA | 21,27 |
| A1 | £338 | GAMMA | 4,21,27 |
| A2 | £5 381 | GAMMA | 4,21,27 |
| B | £4 997 | GAMMA | 21,27 |
| B1 | £338 | GAMMA | 4,21,27 |
| B2 | £4 036 | GAMMA | 4,21,27 |
| C1 | £0 | GAMMA | 4,21,27 |
| C2 | £0 | GAMMA | 4,21,27 |
| *Community weight management intervention arm* | | | |
| A | £624 | GAMMA | 21,27 |
| A1 | £729 | GAMMA | 4,21,27 |
| A2 | £5 919 | GAMMA | 4,21,27 |
| B | £624 | GAMMA | 21,27 |
| B1 | £729 | GAMMA | 4,21,27 |
| B2 | £8 072 | GAMMA | 4,21,27 |
| C1 | £0 | GAMMA | 4,21,27 |
| C2 | £0 | GAMMA | 4,21,27 |
| **Health state costs with additional comorbidities*** | | | |
| *Bariatric surgery arm* | | | |
| A | £4 997 | GAMMA | 21,27 |
| A1 | £1 424 | GAMMA | 4,21,27,31,35 |
| A2 | £6 466 | GAMMA | 4,21,27,31,35 |
| B | £4 997 | GAMMA | 21,27 |
| B1 | £1 159 | GAMMA | 4,21,27,31,35 |
| B2 | £4 856 | GAMMA | 4,21,27,31,35 |
| C1 | £403 | GAMMA | 4,21,27,31,35 |
| C2 | £403 | GAMMA | 4,21,27,31,35 |
| *Community weight management intervention arm* | | | |
| A | £624 | GAMMA | 21,27 |
| A1 | £1 815 | GAMMA | 4,21,27,31,35 |
| A2 | £7 004 | GAMMA | 4,21,27,31,35 |
| B | £624 | GAMMA | 21,27 |
| B1 | £1 550 | GAMMA | 4,21,27,31,35 |
| B2 | £8 892 | GAMMA | 4,21,27,31,35 |
| C1 | £403 | GAMMA | 4,21,27,31,35 |
| C2 | £403 | GAMMA | 4,21,27,31,35 |

*Figure 1 presents the Markov model structure. Reading from left to right there are 3 health states for Y1 (Severe obesity A1; Obesity A1 and No Obesity A3); for Y2-Y10 (Severe obesity B1; Obesity B2; No obesity B3); and for Y11 onwards (Severe obesity C1; Obesity C2; No obesity C3). D denotes Death.

**Table S2: Model transition probabilities**

| **Parameter** | **Value** | **Distribution** | **Source** |
| --- | --- | --- | --- |
| **Transition probabilities*** | | | |
| *Bariatric surgery arm* | | | |
| A-A1 | 0.2837 | BETA | 21 |
| A-B1 | 0.5694 | BETA | 21 |
| A-C1 | 0.1408 | BETA | 21 |
| A-D | 0.0061 | BETA | 21 |
| B-A1 | 0.0000 | BETA | 21 |
| B-B1 | 0.4149 | BETA | 21 |
| B-C1 | 0.5815 | BETA | 21 |
| B-D | 0.0036 | BETA | 21 |
| A1-A2 | 0.7471 | BETA | 21 |
| A1-B2 | 0.2471 | BETA | 21 |
| A1-C2 | 0.0000 | BETA | 21 |
| A1-D | 0.0059 | BETA | 21 |
| B1-A2 | 0.0000 | BETA | 21 |
| B1-B2 | 0.7675 | BETA | 21 |
| B1-C2 | 0.2290 | BETA | 21 |
| B1-D | 0.0035 | BETA | 21 |
| C1-A2 | 0.0000 | BETA | 21 |
| C1-B2 | 0.2213 | BETA | 21 |
| C1-C2 | 0.7768 | BETA | 21 |
| C1-D | 0.0019 | BETA | 21 |
| A2-A3 | 0.9941 | BETA | 21 |
| A2-B3 | 0.0000 | BETA | 21 |
| A2-C3 | 0.0000 | BETA | 21 |
| A2-D | 0.0059 | BETA | 21 |
| B2-A3 | 0.0077 | BETA | 21 |
| B2-B3 | 0.9888 | BETA | 21 |
| B2-C3 | 0.0000 | BETA | 21 |
| B2-D | 0.0035 | BETA | 21 |
| C2-A3 | 0.0000 | BETA | 21 |
| C2-B3 | 0.0268 | BETA | 21 |
| C2-C3 | 0.9713 | BETA | 21 |
| C2-D | 0.0019 | BETA | 21 |
| *Community weight management intervention arm* | | | |
| A-A1 | 0.8152 | BETA | 21 |
| A-B1 | 0.1789 | BETA | 21 |
| A-C1 | 0.0000 | BETA | 21 |
| A-D | 0.0059 | BETA | 21 |
| B-A1 | 0.2710 | BETA | 21 |
| B-B1 | 0.7255 | BETA | 21 |
| B-C1 | 0.0000 | BETA | 21 |
| B-D | 0.0035 | BETA | 21 |
| A1-A2 | 0.9941 | BETA | 21 |
| A1-B2 | 0.0000 | BETA | 21 |
| A1-C2 | 0.0000 | BETA | 21 |
| A1-D | 0.0059 | BETA | 21 |
| B1-A2 | 0.0816 | BETA | 21 |
| B1-B2 | 0.9149 | BETA | 21 |
| B1-C2 | 0.0000 | BETA | 21 |
| B1-D | 0.0035 | BETA | 21 |
| C1-A2 | 0.0000 | BETA | 21 |
| C1-B2 | 0.0000 | BETA | 21 |
| C1-C2 | 0.0000 | BETA | 21 |
| C1-D | 0.0000 | BETA | 21 |
| A2-A3 | 0.9941 | BETA | 21 |
| A2-B3 | 0.0000 | BETA | 21 |
| A2-C3 | 0.0000 | BETA | 21 |
| A2-D | 0.0059 | BETA | 21 |
| B2-A3 | 0.0332 | BETA | 21 |
| B2-B3 | 0.9634 | BETA | 21 |
| B2-C3 | 0.0000 | BETA | 21 |
| B2-D | 0.0035 | BETA | 21 |
| C2-A3 | 0.0000 | BETA | 21 |
| C2-B3 | 0.0000 | BETA | 21 |
| C2-C3 | 0.0000 | BETA | 21 |
| C2-D | 0.0000 | BETA | 21 |

*Figure 1 presents the Markov model structure. Reading from left to right there are 3 health states for Y1 (Severe obesity A1; Obesity A1 and No Obesity A3); for Y2-Y10 (Severe obesity B1; Obesity B2; No obesity B3); and for Y11 onwards (Severe obesity C1; Obesity C2; No obesity C3). D denotes Death.
